# Acute and acute-on-chronic kidney injury of patients with decompensated heart failure: impact on outcomes

**DOI:** 10.1186/1471-2369-13-51

**Published:** 2012-07-02

**Authors:** Qiugen Zhou, Chunmei Zhao, Di Xie, Dingli Xu, Jianping Bin, Pingyan Chen, Min Liang, Xun Zhang, Fanfan Hou

**Affiliations:** 1 Division of Nephrology, Nanfang Hospital, Southern Medical University, Guangzhou, China; 2 Guangdong Provincial Institute of Nephrology, Guangzhou, China; 3 Key Lab for Organ Failure Research, Ministry of Education, Guangzhou, China; 4 Division of Cardiology, Nanfang Hospital, Southern Medical University, Guangzhou, China; 5 Department of biostatistics, School of Public Health and Tropical Medicine, Southern Medical University, Guangzhou, China

**Keywords:** Acute decompensated heart failure, Acute kidney injury, Acute-on-chronic kidney injury, Outcome

## Abstract

**Background:**

Acute worsening of renal function, an independent risk factor for adverse outcomes in acute decompensated heart failure (ADHF), occurs as a consequence of new onset kidney injury (AKI) or acute deterioration of pre-existed chronic kidney disease (CKD) (acute-on-chronic kidney injury, ACKI). However, the possible difference in prognostic implication between AKI and ACKI has not been well established.

**Methods:**

We studied all consecutive patients hospitalized with ADHF from 2003 through 2010 in Nanfang Hospital. We classified patients as with or without pre-existed CKD based on the mean estimated glomerular filtration rate (eGFR) over a six-month period before hospitalization. AKI and ACKI were defined by RIFLE criteria according to the increase of the index serum creatinine.

**Results:**

A total of 1,005 patients were enrolled. The incidence of ACKI was higher than that of AKI. The proportion of patients with diuretic resistance was higher among patients with pre-existed CKD than among those without CKD (16.9% *vs.* 9.9%, P = 0.002). Compared with AKI, ACKI was associated with higher risk for in-hospital mortality, long hospital stay, and failure in renal function recovery. Pre-existed CKD and development of acute worsening of renal function during hospitalization were the independent risk factors for in-hospital death after adjustment by the other risk factors. The RIFLE classification predicted all-cause and cardiac mortality in both AKI and ACKI.

**Conclusions:**

Patients with ACKI were at greatest risk of adverse short-term outcomes in ADHF. Monitoring eGFR and identifying CKD should not be ignored in patients with cardiovascular disease.

## Background

Acute decompensated heart failure (ADHF) is one of the leading causes of hospitalization worldwide. More than 70% of patients hospitalized for ADHF will experience acute worsening of renal function, which is associated with significantly poor outcomes [1-7]. Patients with ADHF are commonly accompanied by the co-morbidities such as hypertension, diabetes mellitus, and atherosclerosis which are the risk factors for chronic kidney disease (CKD). The Acute Decompensated Heart Failure National Registry, a large database of patients with ADHF requiring hospitalization in the United States, reported that 30% had an additional diagnosis consistent with CKD [8]*.* Therefore, acute worsening of renal function in ADHF might be a consequence of new onset kidney injury (AKI) or acute deterioration of pre-existed CKD (acute-on-chronic kidney injury, ACKI).

Increasing evidence has shown that CKD contributes to impairment of cardiovascular structures and function [9]. Thus, patients with AKI and ACKI may have different impact on outcomes and unique responses to therapeutic regimens. However, few studies have done to compare the clinical characteristics between AKI and ACKI, particularly the impact on outcomes. It remains unclear whether worsening renal function specifically contributes to adverse outcomes or whether it merely serves as a marker of advanced cardiac/or renal dysfunction.

The present study was performed to compare the impact on outcomes of AKI and ACKI in a cohort of 1,005 Chinese patients with ADHF. We aimed to test the hypotheses that patients with ACKI, as opposed to those with AKI, may be at greater risk of adverse outcomes during hospitalization in the setting of ADHF.

## Methods

The study was approved by the Review Board of Nanfang hospital. A total of 1,230 patients with ADHF were hospitalized to the Coronary Care Unit (CCU) in Nanfang hospital, Guangzhou, between Jan 1, 2003, and Dec 31, 2010. Data on estimated glomerular filtration rate (eGFR) before admission were available for 1,005 of these patients (81%).

### Identification of patients

The integrated medical record system of the hospital, identified each patient with a unique number, served as the basis for our retrospective analysis. The diagnosis of ADHF was based on European Society of Cardiology Criteria [10]. The patients discharged with the diagnosis codes of heart failure according to the International Classification of Disease, Ninth Revision, Clinical Modification [11], were considered for inclusion in the study. If a patient was hospitalized more than once for ADHF during the study period, only the data from the first admission were analyzed.

The exclusion criteria included severe aortic stenosis, pulmonary thromboembolism, cardiac tamponade, cardiogenic shock, heart failure following cardiac surgery, or multi-organ failure. Patients were also excluded if they had chronic and severe renal failure (chronic dialysis or eGFR below 30 ml/min/1.73 m^2^ before admission) or worsening of renal function occurred following surgery or administration of potentially nephrotoxic agents such as contrast medium. Subjects who had no records of serum creatinine values over 6-month period before admission or during hospital were not included in the study.

### Data extraction

Data were collected on the patient’s demographic characteristics and clinical manifestations on admission. Data on coexisting cardiovascular conditions in each patient were also extracted with the use of all relevant ICD codes. Data on laboratory analysis were extracted from the Laboratory Information System of Nanfang hospital.

To verify the accuracy of chart abstraction, an independent abstractor re-evaluated information in four categories (creatinine, inclusion and exclusion criteria, and discharge dates). Serum creatinine values for all 1,005 patients in the final study population were checked, and no discrepancies were detected. In addition, comprehensive examinations of all data fields were completed in a subset of 10% of the subjects. Less than 0.5% discrepancy was detected.

### Definition of covariates and category

We identified all patients with mean eGFR (at least 3 measurements) more than 30 ml/min/1.73 m^2^ over a 6-month period before admission. This eGFR value was termed the “index eGFR”. We estimated the GFR using the simplified Modification of Diet in Renal Disease (MDRD) equation [12], which is accepted as a valid method for estimating glomerular filtration in patients with heart failure [13]. We also estimated the GFR with the Chronic Kidney Epidemiology Collaboration equation (CKD-EPI) which has been shown to be more accurate in various populations including Asian [14,15]. The results obtained from the two equations were comparable. Thus, eGFR values in the present study were expressed as that calculated with CKD-EPI equation. Since creatinine is not in steady state when AKI occurs, it is not appropriate to calculate the GFR from serum creatinine [16]. We used the peak increase in serum creatinine to assign a category in the RIFLE classification. We did not use urine output as a criterion for classification, because it was not possible to obtain accurate records of urine output.

#### AKI group

Patients with index eGFR were classified as having AKI when their serum creatinine was increased by 50%, 100%, or 200% during hospitalization. 93% of patients in the cohort reached their peak serum creatinine within the first 7 days of hospitalization. We used maximum serum creatinine level to category RIFLE class.

#### ACKI group

The Acute Dialysis Quality Initiative (ADQI) group recommends separate criteria for the diagnosis of ACKI. They assigned these patients to the Fc category (where F is failure and c is chronic kidney disease) when their serum creatinine had increased to 350 μmol/L. The ADQI group did not assign a category to those in whom serum creatinine did not rise as high as 350 μmol/L. Patients in our study were defined as CKD when their index eGFR values were arrange from 60 ml/min/1.73 m^2^ to 30 ml/min/1.73 m^2^. We excluded patients with eGFR below 30 ml/min/1.73 m^2^, since it may be difficult to distinguish between the final stages of progression to end stage renal disease and potentially reversible acute worsening of renal function due to relatively small changes in GFR leading to large changes in serum creatinine [17].

We used the following classification of ACKI in this study according to previous report [17]:

1. Risk: serum creatinine increased by 50% or more from index serum creatinine but had not reached 350 μmol/L.

2. Injury: serum creatinine increased by 100% or more from index serum creatinine but had not reached 350 μmol/L.

3. Failure: serum creatinine increased by 200% or more from index serum creatinine or serum creatinine had increased to 350 μmol/L as the ADQI group recommends.

Patients were excluded when any rise in serum creatinine was not sustained for 24 hours.

#### Definition of renal recovery

Renal recovery was defined as previously reported [17].

1. Full recovery: Serum creatinine concentrations fell below or to the index .

2. Partial recovery: Serum creatinine remained above the index.

3. Failure to recover: Dialysis dependent at 90 day.

#### Diuretic resistance

Diuretic resistance was defined as persistent pulmonary congestion with or without acute worsening of renal function despite attempts at diuresis (repeated doses of 80 mg furosemide, or greater than 240 mg furosemide daily, or combination diuretic therapy including loop diuretics with thiazide or aldosterone antagonist). The doses of diuretics were calculated according to the prescription records in chart review.

### Statistical analysis

The continuous variables were presented as the mean ± standard deviation or the median and interquartile ranges where appropriate. Categorical variables are presented as percentages or proportion. For the univariate analysis, we compared two groups using the Student’s *t* test when normally distributed, and the Mann–Whitney test when not. The Pearson *χ*^2^ test and the Kruskal-Wallis test were applied for analysis of nominal and ordinal variables, respectively.

The univariate analysis was conducted to screen the risk factors at a significant level of 0.20. Multivariate logistic regression analysis was performed to assess the impact of AKI and ACKI on the all-cause and cardiovascular in-hospital mortality. The marked independent risk factors of mortality were identified by stepwise method of Wald’s forward selection. Adjusted odds ratio and the 95% confidence interval for each notable risk factor in the model were derived. Similarly, the risk factors for development of acute worsening of renal function were investigated using variables obtained before the occurrence of acute renal injury. Model calibration was assessed by using the Hosmer–Lemeshow goodness-of-fit test. The variance inflation factor (VIF) was used for detecting the co-linearity and a VIF of 10 and above indicates a co-linearity problem. All tests were two-tailed and P < 0.05 was considered significant. Data were analyzed using SPSS 13.0 for Windows^®^.

## Results

### Comparison of clinical characteristics between AKI and ACKI

A total of 1,005 patients were included in the study. Among them, 738 patients had index eGFR equal or above 60 ml/min/1.73 m^2^ and 267 patients had CKD with index eGFR 30–59 ml/min/1.73 m^2^. The characteristics and comparison between two groups are listed in Table 1. Compared with patients with preserved renal function, the patients with CKD were older and had more coexisted diseases such as diabetes mellitus, hypertension, ischemic heart disease and cerebrovascular disease. Patients with CKD had lower concentration of serum albumin and hemoglobin and higher levels of blood pressure compared with patients without CKD.

**Table 1:** Characteristics of patients classified by the index eGFR

	**Total cohort (n = 1005)**	**Index eGFR, ml/min/1.73 m**^**2**^		
		**≥60 (n = 738)**	**30 ~ 59 (n = 267)**	***P***
**Demographics**				
Age, years	63 ± 16	60 ± 16	71 ± 12	<0.001
Male, no. (%)	625(62.2)	461(62.5)	164(61.4)	0.763
Current smoker, no. (%)	317(31.5)	240(32.5)	77(28.8)	0.267
**Comorbid conditions**				
Diabetes, no. (%)	368(36.6)	243(32.9)	125(46.8)	<0.001
Hypertension, no. (%)	485(48.3)	304(41.2)	181(67.8)	<0.001
Ischemic heart disease, no. (%)	506(50.3)	354(48.0)	152(56.9)	0.012
Atrial fibrillation, no. (%)	288(28.7)	217(29.4)	71(26.6)	0.384
Cerebrovascular disease, no. (%)	112(11.1)	71(9.6)	41(15.4)	0.011
Comorbid sum ^a^, no. (%)				<0.001
0	118(11.7)	104(14.1)	14(5.2)	
1	305(30.3)	250(33.9)	55(20.6)	
2	347(34.5)	242(32.8)	105(39.4)	
≥3	235(23.4)	142(19.2)	93(34.9)	
**Index eGFR, ml/min/1.73 m**^**2**^	78 ± 25	89 ± 18	47 ± 9	<0.001
**Characteristics on admission**				
LVEF < 45%, no. (%)	417(41.5)	305(41.3)	112(41.9)	0.860
NYHA class 4, no. (%)^b^	467(46.5)	315(42.7)	152(56.9)	<0.001
Systolic blood pressure, mm Hg	130 ± 20	127 ± 19	136 ± 23	<0.001
Diastolic blood pressure, mm Hg	80 ± 22	79 ± 18	84 ± 30	0.002
Serum creatinine, μmol/L	110 ± 64	90 ± 31	164 ± 95	<0.001
Fasting plasma glucose, mmol/L	6.5 ± 2.3	6.4 ± 2.3	6.6 ± 2.6	0.385
Serum triglyeride, mmol/L	1.5 ± 1.4	1.4 ± 1.3	1.6 ± 1.6	0.353
Serum total cholesterol, mmol/L	4.6 ± 1.4	4.6 ± 1.4	4.6 ± 1.3	0.579
Serum LDL-C, mmol/L	2.3 ± 1.0	2.4 ± 1.0	2.3 ± 1.0	0.564
Serum albumin, g/L	36.2 ± 5.6	36.7 ± 5.2	34.6 ± 6.2	<0.001
Haemoglobin, g/L	128 ± 23	131 ± 22	118 ± 24	<0.001
**Inotropic therapy, no. (%)**	134(13.3)	104(14.1)	30(11.2)	0.239

^a^ Number of comorbid conditions in a patient.

^b^ NYHA class captured according to the manifestation during the first day of hospitalization.

**Abbreviation:** AHF, acute heart failure; eGFR, estimated glomerular filtration rate; LDL-C, low density lipoprotein cholesterol; LVEF, left ventricular ejection fraction; NYHA, New York Heart Association.

According to the RIFLE criteria, acute worsening of renal function occurred in 445 (44.3%) patients of the cohort. As shown in Table 2, the incidence of acute worsening of renal function was higher among patients with pre-existed CKD than among those without. Additionally, the proportion of patients with severe acute worsening of renal function (injury and failure category) and those who need renal replacement therapy (RRT) were more prevalent in CKD group. Notably, 19 patients in ACKI group received RRT, only 9 patients were in Failure category. Ten patients treated with RRT due to fluid overload (n = 6), hyperkalemia (n = 2) or severe acidosis (n = 2).

**Table 2:** The characteristics of acute worsening of renal function in patients classified by the index eGFR

	**Total**	**Index eGFR, ml/min/1.73 m**^**2**^		
		**≥60**	**30 ~ 59**	***P***
**AWRF incidence, n/N (%)**	445/1005 (44.3)	294/738 (39.8)	151/267 (56.6)	<0.001
**RIFLE category of AWRF, n/N (%)**				<0.001
Risk	282/445 (63.4)	204/294 (69.4)	78/151 (51.7)	
Injury	127/445 (28.5)	63/294 (21.4)	64/151 (42.4)	
Failure	36/445(18.1)	27/294 (9.2)	9/151 (6.0)	
**Maximum serum creatinine during hospitalization, μmol/L**	182 ± 118	145 ± 73	254 ± 152	<0.001
**Median time of maximum RIFLE class reached**^a^**, day**	4 (1 ~ 8)	4(1 ~ 8)	4(2 ~ 8)	0.118
**RRT required, n/N (%)**	25/445 (5.6)	6/294 (2.0)	19/151 (12.6)	<0.001
**Diuretic resistance, n/N (%)**	88/445(19.8)	52/294(17.7)	36/151(23.8)	0.123
**Ultrafitration for diuretic resistance, n/N (%)**	19/445(4.3)	6/294 (2.0)	13/151 (8.6)	0.002

^a^ Values expressed as median (25th percentile -75th percentile).

**Abbreviation:** AWRF, acute worsening of renal function; eGFR, estimated glomerular filtration rate; RIFLE, risk, injury, failure, loss, end-stage renal disease; RRT, renal replacement therapy.

Since diuretic resistance is the most extreme manifestation of ADHF and associated with the adverse outcomes [18], we compared the incidence of diuretic resistance during hospitalization in patients with and without pre-existed CKD. As shown in Table 2, diuretic resistance seemed more prevalent in patients with ACKI than those with AKI (23.8% *vs.* 17.7%), although the difference did not reach statistical significance. However, more patients with ACKI needed ultrafitration for diuretic resistance compared to those with AKI.

### Impact of AKI and ACKI on outcomes

#### In-hospital mortality and length of stay

In-hospital outcomes in patients classified by the index eGFR were shown in Table 3. In patients with AKI, the all-cause in hospital mortality was 16.7% and the cardiovascular mortality was 11.9%. While in those with ACKI, the all-cause in hospital mortality was 24.5% and the cardiovascular mortality was 23.2%. The incidence of all-cause as well as cardiac death was significantly higher in patients with ACKI than those with AKI (P < 0.05 in all). Acute worsening of renal function during hospitalization significantly increased all-cause and cardiac death in both AKI and ACKI (Table 3). With respect to patients with acute worsening of renal function, there was a stepwise increase in the incidence of all-cause and cardiac mortality from RIFLE category risk to category failure in both AKI and ACKI (Table 3).

**Table 3:** In-hospital outcomes in patients with acute worsening of renal function

	**Index eGFR**											
	**≥60 ml/min/1.73 m**^**2**^						**30 ~ 59 ml/min/1.73 m**^**2**^					
	**No AWRF** (n = 444)	**AKI group**					**No AWRF** (n = 116)	**ACKI group**				
		All (n = 294)	Risk (n = 204)	Injury (n = 63)	Failure (n = 27)	P ^c^		All (n = 151)	Risk (n = 78)	Injury (n = 64)	Failure (n = 9)	P ^c^
**In-hospital mortality**												
All cause mortality, no. (%)	23(5.2)	49(16.7) ^a^	15(7.4)	16(25.4)	18(66.7)	<0.001	11(9.5)	37(24.5) ^a, b^	11(14.1)	21(32.8)	5(55.6)	0.003
Cardiovascular mortality, no. (%)	17(3.8)	35(11.9) ^a^	12(5.9)	11(17.5)	12(44.4)	<0.001	8(6.9)	35(23.2) ^a, b^	11(14.1)	19 (29.7)	5 (55.6)	0.005
**Outcomes of survivors**												
Length of stay in CCU, days	3(2 ~ 4)	3(2 ~ 4) ^a^	3(2 ~ 4)	3(2 ~ 4)	4(3 ~ 9)	0.001	4(2 ~ 4)	5(4 ~ 6) ^a,b^	4(4 ~ 5)	5(4 ~ 6)	7(4 ~ 7)	0.027
Length of stay in hospital, days	8(6 ~ 14)	14(9 ~ 18) ^a^	12(8 ~ 17)	17(12 ~ 27)	20(15 ~ 33)	<0.001	8(6 ~ 13)	15(11 ~ 22) ^a, b^	14(8 ~ 18)	18(14 ~ 23)	20(9 ~ 32)	0.001

^a^*P* < 0.05 *vs.* patients without AWRF; ^b^*P* < 0.05 *vs.* patients with AKI; ^c^*P* for comparison among RIFLE category.

**Abbreviation**: ACKI, acute-on-chronic kidney injury; AKI, acute kidney injury; AWRF, acute worsening of renal function; CCU, coronary care unit; eGFR, estimated glomerular filtration rate.

Development of acute worsening of renal function significantly prolonged the length of stay in CCU as well as in hospital. The severity of AKI defined by RIFLE category correlated with length of stay in CCU and hospital. A similar trend was observed among patients with ACKI.

#### Renal recovery

Full renal recovery was achieved in 177 (72.3%) of those in AKI group at discharge, 67 (27.3%) partially recovered, and 1 (0.4%) failed to recover. In contrast, in ACKI group, full recovery was achieved in only 35 (30.7%) at discharge, partial recovery was achieved in 73 (64.0%), and 6 (5.3%) remained on dialysis at 90 days.

**Figure 1:**
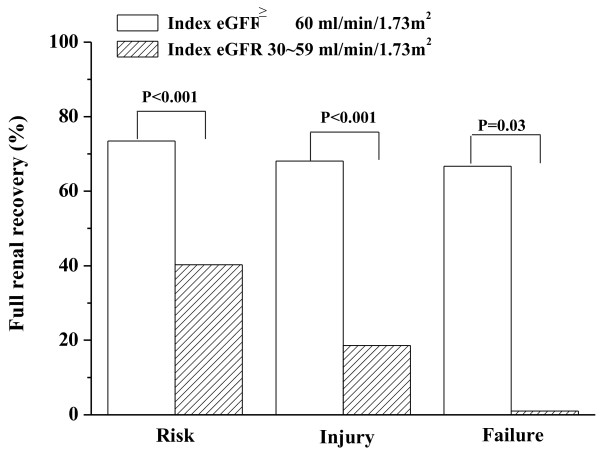
**Full renal recovery rate at discharge in patients with AKI and ACKI stratified by RIFLE criteria.** At any level of acute worsening of renal function, ACKI group had less proportion of full renal recovery as compared with AKI group.

As shown in Figure 1, at any level of acute worsening of renal function, ACKI group had significantly less proportion of renal recovery as compared with AKI group.

#### Re-hospitalization

Re-hospitalization rate also represents an important outcome. As shown in Table 4, one-year re-hospitalization for subsequent ADHF and total reasons after the index hospitalization was significantly higher in both AKI and ACKI compared to those with ADHF alone. Notably, development of ACKI during the index hospitalization significantly increased subsequent one-year re-hospitalization for both ADHF and total reasons as compared with those with AKI.

**Table 4:** One-year re-hospital rate in survival patients with acute worsening of renal function

	**Index eGFR**					
	**≥60 ml/min/1.73 m**^**2**^			**30 ~ 59 ml/min/1.73 m**^**2**^		
	**No AWRF** **(n = 421)**	**AKI group** **(n = 245)**	***P***	**No AWRF** **(n = 105)**	**ACKI group** **(n = 114)**	***P***
**Total re-hospitalization, n (%)**	80 (19.0)	64 (26.1)	0.031	19 (18.1)	38 (33.3)	0.010
For ADHF	16 (3.8)	22 (9.0)	0.005	3 (2.9)	16 (14.0)	0.003
For other reasons	64 (15.2)	42 (17.1)	0.509	16 (15.2)	22 (19.3)	0.428

**Abbreviation:** ACKI, acute-on-chronic kidney injury; ADHF, acute decompensated heart failure; AKI, acute kidney injury; AWRF, acute worsening of renal function; eGFR, estimated glomerular filtration rate.

### The factors correlated with the outcomes of AKI and ACKI

To identify the factors correlated with in-hospital mortality, we conducted univariate and multivariate logistic regression analysis. No co-linearity was found between variables. Table 5 showed adjusted odd ratios for all-cause mortality in the total cohort and in the subgroups classified by index eGFR. Pre-existed CKD and development of acute worsening of renal function during hospital were still the independent risk factors for all-cause mortality in total cohort after adjustment by age, gender, smoking, blood pressure, LDL or HDL cholesterol, serum albumin, hemoglobin, co-morbid diseases, and renal function (serum creatinine and eGFR) on admission.

**Table 5:** Multivariate logistic regression analysis: risk factors for all-cause mortality

	**Total**^**a**^		**Index eGFR (ml/min/1.73 m**^**2**^**)**			
			**≥60**^**b**^		**30 ~ 59**^**c**^	
	**OR (95% CI)**	***P***	**OR (95% CI)**	***P***	**OR (95% CI)**	***P***
**Cerebrovascular disease (yes*vs.*no)**	1.86 (1.05 ~ 3.29)	0.034	-	-	-	-
**Index eGFR < 60 ml/min/1.73 m**^**2**^**(yes*vs.*no)**	1.66 (1.06 ~ 2.62)	0.028	NA	NA	NA	NA
**RIFLE category of AWRF (*vs.*no AWRF)**	1.82 (1.14 ~ 2.90)	0.013	-	<0.001	-	0.001
Risk	-	-	0.88 (0.43 ~ 1.79)	0.715	0.70 (0.26 ~ 1.91)	0.486
Injury	-	-	4.20 (1.96 ~ 9.01)	<0.001	3.32 (1.36 ~ 8.08)	0.008
Failure	-	-	19.87 (6.35 ~ 44.79)	<0.001	7.38(1.50 ~ 36.37)	0.014
**LVEF < 45% on admission (yes*vs.*no)**	2.16 (1.40 ~ 3.35)	0.001	1.88(1.07 ~ 3.31)	0.028	4.05 (1.91 ~ 8.60)	<0.001
**NYHA class 4 on admission (yes*vs.*no)**	2.55 (1.55 ~ 4.20)	<0.001	2.07(1.10 ~ 3.90)	0.024	5.50 (2.07 ~ 14.62)	<0.001
**Systolic blood pressure > 160 mmHg on admission (yes*vs.*no)**	2.30 (1.05 ~ 5.08)	0.039	-	-	-	-
**Fasting plasma glucose > 7.0 mmol/L on admission (yes*vs.*no)**	3.93 (2.54 ~ 6.07)	<0.001	-	-	-	-
**Diuretic resistance (yes*vs.*no)**	2.83 (1.72 ~ 4.64)	<0.001	3.99 (2.01 ~ 7.90)	<0.001	2.71 (1.17 ~ 6.28)	0.020

^a^ Hosmer-Lemeshow goodness-of-fit test: chi-square value = 3.441, P = 0.841.

^b^ Hosmer-Lemeshow goodness-of-fit test: chi-square value = 2.970, P = 0.888.

^c^ Hosmer-Lemeshow goodness-of-fit test: chi-square value = 5.355, P = 0.719.

**Abbreviation**: AWRF, acute worsening of renal function; CI, confidence interval; eGFR, estimated glomerular filtration rate; LVEF, left ventricular ejection fraction; NYHA, New York Heart Association; OR, odd ratio;RIFLE, risk, injury, failure, loss, end-stage renal disease.

Likewise, severe acute worsening of renal function (RIFLE category Injury or Failure) was one of the strongest risk factors in ADHF patients with or without pre-existed CKD when the analysis was separately conducted in the subgroups. Severe heart failure (NYHA class 4) and diuretic resistance were significant risk factors for mortality after adjustment by the above variables. Similar risk factors have been identified for cardiovascular mortality in the cohort (Table 6).

**Table 6:** Multivariate logistic regression analysis: risk factors for cardiovascular mortality

	**Total**^**a**^		**Index eGFR (ml/min/1.73 m**^**2**^**)**			
			**≥60**^**b**^		**30 ~ 59**^**c**^	
	**OR (95% CI)**	***P***	**OR (95% CI)**	***P***	**OR (95% CI)**	***P***
**Index eGFR < 60 ml/min/1.73 m**^**2**^**(yes*vs.*no)**	2.21 (1.37 ~ 3.58)	0.001	NA	NA	NA	NA
**RIFLE category of AWRF (*vs.*no AWRF)**	1.95 (1.16 ~ 3.29)	0.012	-	<0.001	-	<0.001
Risk	-	-	1.06 (0.48 ~ 2.33)	0.890	1.07 (0.37 ~ 3.08)	0.895
Injury	-	-	3.49 (1.48 ~ 8.26)	0.004	4.09 (1.56 ~ 10.70)	0.004
Failure	-	-	10.00 (3.74 ~ 26.76)	<0.001	10.76 (2.12 ~ 54.48)	0.004
**LVEF < 45% on admission (yes*vs.*no)**	2.17 (1.35 ~ 3.51)	0.002	2.02(1.08 ~ 3.77)	0.028	3.37(1.55 ~ 7.30)	0.002
**NYHA class 4 on admission (yes*vs.*no)**	2.58 (1.48 ~ 4.50)	0.001	2.03 (1.02 ~ 4.06)	0.045	5.49 (1.92 ~ 15.71)	0.001
**Systolic blood pressure > 160 mm Hg on admission (yes*vs.*no)**	2.74 (1.22 ~ 6.17)	0.015	-	-	-	-
**Fasting plasma glucose > 7.0 mmol/L on admission (yes*vs.*no)**	3.98 (2.46 ~ 6.43)	<0.001	-	-	-	-
**Diuretic resistance (yes*vs.*no)**	2.03 (1.19 ~ 3.49)	0.010	2.21(1.03 ~ 4.74)	0.042	2.69(1.15 ~ 6.25)	0.022

^a^ Hosmer-Lemeshow goodness-of-fit test: chi-square value = 10.833, P = 0.146.

^b^ Hosmer-Lemeshow goodness-of-fit test: chi-square value = 2.723, P = 0.843.

^c^ Hosmer-Lemeshow goodness-of-fit test: chi-square value = 5.681, P = 0.577.

**Abbreviation**: AWRF, acute worsening of renal function; CI, confidence interval; eGFR, estimated glomerular filtration rate; LVEF, left ventricular ejection fraction; NYHA, New York Heart Association; OR, odd ratio;RIFLE, risk, injury, failure, loss, end-stage renal disease.

Multivariate logistic regression analysis was also performed to identify the risk factors for development of acute worsening of renal function. As shown in Table 7, the independent risk factors for acute worsening of renal function in ADHF were diabetes, pre-existed CKD, systolic hear failure (LVEF < 45%) or severe heart failure (NYHA class 4), severe systolic hypertension (systolic blood pressure > 160 mmHg), and diuretic resistance.

**Table 7:** Multivariate logistic regression analysis: risk factors for development of acute worsening of renal function

	**Total**^**a**^		**Index eGFR (ml/min/1.73 m**^**2**^**)**			
			**≥60**^**b**^		**30 ~ 59**^**c**^	
	**OR (95% CI)**	***P***	**OR (95% CI)**	***P***	**OR (95% CI)**	***P***
**Diabetes (yes*vs.*no)**	2.00(1.50 ~ 2.66)	<0.001	1.74(1.24 ~ 2.43)	0.001	2.62(1.50 ~ 4.57)	0.001
**Index eGFR < 60 ml/min/1.73 m**^**2**^**(yes*vs.*no)**	1.54 (1.13 ~ 2.11)	0.007	NA	NA	NA	NA
**LVEF < 45% on admission (yes*vs.*no)**	1.73 (1.31 ~ 2.29)	<0.001	1.59(1.15 ~ 2.20)	0.005	2.39(1.36 ~ 4.20)	0.002
**NYHA class 4 on admission (yes*vs.*no)**	3.10 (2.35 ~ 4.10)	<0.001	3.53(2.55 ~ 4.89)	<0.001	2.40(1.38 ~ 4.18)	0.002
**Systolic blood pressure > 160 mmHg on admission (yes*vs.*no)**	3.80 (1.67 ~ 8.49)	0.001	4.07(1.23 ~ 13.42)	0.021	4.04(1.31 ~ 12.44)	0.015
**Diuretic resistance (yes*vs.*no)**	2.09 (1.55 ~ 2.81)	<0.001	1.70(1.20 ~ 2.41)	0.003	3.63 (1.94 ~ 6.78)	<0.001

^a^ Hosmer-Lemeshow goodness-of-fit test: chi-square value = 4.722, P = 0.694.

^b^ Hosmer-Lemeshow goodness-of-fit test: chi-square value = 2.437, P = 0.875.

^c^ Hosmer-Lemeshow goodness-of-fit test: chi-square value = 7.809, P = 0.452.

**Abbreviation:** CI, confidence interval; eGFR, estimated glomerular filtration rate; LVEF, left ventricular ejection fraction; NA, not analysis; NYHA, New York Heart Association; OR, odd ratio.

Therapeutics approaches for management of HF might also influence the outcomes. In our cohort, only 10 patients received nesiritide treatment, probably due to exclusion of those with cardiac shock before entry. A few patients had been treated with cardiac resynchronization (n = 20) or left-ventricular assist devices (n = 9). Therefore, we were not able to analyze the effect of these approaches on the outcomes because of the limited number of cases.

## Discussion

Acute worsening of renal function in patients with ADHF is common and increasingly recognized as an independent risk factor for morbidity and mortality [19-23]. Renal impairment in ADHF develops as a consequence of new onset kidney injury (AKI) or of acute deterioration of pre-existed CKD (ACKI). This study is the first study, to the best of our knowledge, comparing the prognostic implications of AKI with ACKI. The results demonstrated that acute worsening of renal function in ADHF was more prevalent in patients with pre-existed CKD than those without. Patients with ACKI, as opposite to those with AKI, were at greater risk of adverse outcomes during hospitalization for ADHF.

The reported incidence of acute worsening of renal function in ADHF varies from 29% to 70% depending on the study entry criteria and the definition used to characterize renal dysfunction [3,24]. In our study, despite exclusion of cardiogenic shock, contrast medium-induced nephropathy, and severe chronic renal failure, acute worsening of renal function was still very frequent (44.3%) during hospitalization for ADHF. The incidence was similar with the previous study which enrolled patients with similar characteristics [25]. In contrast to the most previous studies [2-4,23,26] in which serum creatinine on admission or back-calculating creatinine was used as the baseline renal function, we applied preadmission eGFR as the underlying renal function and defined AKI and ACKI without bias [27]. An important finding in our results was that ACKI was more frequent than AKI (56.6% *vs.* 39.8%) in ADHF. Compared with AKI, ACKI was associated with more severe renal injury (defined as RIFLE category) and more proportion of patients in this group required renal replacement therapy during hospitalization. Consistent with the previous report [24], worsening renal function occurred relatively early in the course of the hospitalization with the median time of 4 days of maximum RIFLE class reached.

Diuretic resistance is common in ADHF, while its prevalence and prognostic implications are less well defined. It is noteworthy that diuretic resistance in our cohort was more prevalent among those with worsening renal function (23.8% in ACKI and 17.7% in AKI) than those with ADHF alone (7.8% in those with pre-existed CKD and 4.7% in patients without CKD). In patients with worsening renal function, prevalence of diuretic resistance seemed to be higher in ACKI than that in AKI group. Although the mechanisms underlying diuretic resistance remain to be clarified, it has been suggested that hypoalbuminemia, commonly seen in CKD, may increase the volume distribution of loop diuretics and impair their delivery to the kidney [24]. Moreover, accumulation of organic acids in CKD may act indirect competition with diuretics for secretion at the proximal tubule [28]. Given the fact that diuretic resistance, particularly with worsening renal function, results in marked persistent volume over-load in ADHF [24], it may represents a subset of more advanced HF and contribute to the poor outcomes. In this study, we demonstrated that more patients with ACKI needed ultrafitration for diuretic resistance compared to those with AKI. The presence of diuretic resistance was identified as one of the strongest independent risk factors for all-cause and cardiac mortality in patients with ADHF, particularly those with pre-existed CKD (Table 4 &5).

Renal dysfunction is one of the most important risk factors for poor outcomes in patients with ADHF [29,30]. However, the difference in prognostic implications between AKI and ACKI has not been well established. Our results demonstrated that patients with ACKI were at higher risk of all-cause and cardiac mortality than those with AKI. Among survivors in the cohort, those with ACKI had longer hospital and CCU stay and higher re-hospitalization as compared with AKI patients. Consistent with the previous study [31], the severity of acute kidney injury predicted non-renal recovery, particularly in patients with AKI. The patients with pre-existed CKD were older, and had more co-morbid diseases such as diabetes, hypertension and ischemic heart disease. More proportion of those with background CKD had lower levels of serum albumin and hemoglobin. To verify the impact of ACKI and AKI on outcomes, logistic regression was used to adjust for the possible confounding factors. In addition to known risk factors, pre-existed CKD and acute worsening of renal function during hospitalization were still found to be significantly associated with all-cause and cardiac mortality. Supporting with our results, in a community-based cohort of patients with CKD, an episode of superimposed dialysis-requiring ARF was associated with very high risk for non-recovery of renal function [32]. In the multivariate logistic regression analysis, we also found that pre-existed CKD was an independent risk factor for development of acute worsening of renal function during admission. When the patients who died in hospital were excluded, full recovery of renal function at discharge was 72.2% in those with AKI, which was much lower than that in the population–base studies (93%) [17] and also lower than that in patients with post-trauma AKI (77.5%) [33]. It is noteworthy, creatinine values were restored to previous levels in only 30.7% of those who had pre-existed CKD and survived their acute illness, though we could not exclude the possibility that the relatively low rate of renal recovery might be related to the strict definition for full renal recovery used in the study.

RIFLE classification provides a well-stratification system for acute renal injury and has been used more commonly [34]. In our study, the RIFLE was able to predict all-cause and cardiac mortality in both AKI and ACKI. The predictive effect was still significant after adjustment by the confounding factors, suggesting that RIFLE classification might be useful for stratification of patients with concomitant cardiac and renal dysfunction. Consistent with early report [35], the RIFLE criteria was suitable to evaluate the AKI, as well as to predict its association with adverse outcome in patients with ADHF.

Acute worsening of renal function in patients with ADHF has been described as cardiorenal syndrome type 1 [36]. The previous reports studied the syndrome have not differentiated AKI and ACKI. Our results indicated that ACKI, as compared with AKI, was associated with higher risk of adverse outcomes, suggesting that type 1 cardiorenal syndrome should be classified in two subgroups based on the underlying renal function. Since the prevalence of CKD has been increasing, particularly in those with cardiovascular disease, it is important to identify CKD early. Estimated GFR should be included in the assessment of risk stratification for individual patients with cardiovascular disease, in addition to traditional cardiovascular risk factors. Since ACKI increased mortality and treatment cost, the need for adequate definition and early screening has never been greater.

We classified ACKI based on RIFLE criteria. This classification may have missed a significant number of patients because those with preexisted CKD would require a considerable increase in creatinine to enter this classification (e.g., a baseline creatinine of 200 μmol/L requires a rise to 300 μmol/L for entry into the R category). Appropriate criteria for ACKI therefore needs further study and definition.

## Conclusion

Our study compared the prognostic implications between AKI and ACKI in a cohort of 1,005 Chinese patients with ADHF. The results showed that ACKI was more frequent than AKI in ADHF. As compared with AKI, ACKI was associated with higher risk of in-hospital mortality, diuretic resistance, prolonged hospital stay, and failure in renal recovery. RIFLE classification predicted all-cause and cardiac mortality in both AKI and ACKI, making it useful to stratify the patients with concomitant cardiac and renal dysfunction.

## Competing interests

The authors declare no conflicts of interests.

## Authors’ contributions

QZ and CZ were involved in the study design, sample collection, and participated in the interpretation of the data and in the writing of the report. DX participated in the analysis and interpretation of the data. DX, JB acquired the data. PC participated in the analysis of the data. ML and XZ participated in the sample collection. FH was involved in the study design, analysis and interpretation of the data and in the writing of the report. All authors have read and approved the final version of the manuscript.

## Pre-publication history

The pre-publication history for this paper can be accessed here:

http://www.biomedcentral.com/1471-2369/13/51/prepub
